# Long-Term Prognostic Value of Coronary Computed Tomography Angiography

**DOI:** 10.1007/s11936-017-0588-5

**Published:** 2017-10-12

**Authors:** Takor B. Arrey-Mbi, Seth M. Klusewitz, Todd C. Villines

**Affiliations:** Cardiology Service, Department of Medicine, Walter Reed National Military Medical Center, 8930 Brown Drive, Bld 9A, Room 2335, Bethesda, MD 20889 USA

**Keywords:** Atherosclerosis, Cardiac CT, Coronary artery disease, Coronary CT angiography, Prognosis

## Abstract

Coronary CT angiography (CTA) is a highly accurate test for the diagnosis of coronary artery disease (CAD), with its use guided by numerous contemporary appropriate use criteria and clinical guidelines. Unique among non-invasive tests for CAD, coronary CTA provides direct visualization of coronary atherosclerosis for the assessment of angiographic stenosis, as well as validated measures of plaque vulnerability. Long-term studies now clearly demonstrate that the absence of CAD on coronary CTA identifies a patient that is at very low risk for future cardiovascular events. Conversely, the presence, location, and severity of CAD as measured on coronary CTA provide powerful prognostic information that is superior to traditional risk factors and other clinical variables. Observational studies and data obtained from clinical trials suggest that the anatomic information derived from coronary CTA significantly increases the utilization of statins and aspirin. Furthermore, these changes are associated with reductions in the risk for mortality, revascularizations, and incident myocardial infarctions among subjects with coronary atherosclerosis. As a result, current societal consensus statements have attempted to standardize coronary CTA reporting, to include incorporation of vulnerable plaque features and recommendations on the use of preventive therapies, such as statins, so to more consistently link important prognostic findings on coronary CTA to appropriate preventive and therapeutic interventions. Automated measures of total coronary plaque volume, machine learning, and CT-derived fractional flow reserve may further refine the prognostic accuracy of coronary CTA. Herein, we summarize recently published literature that reports the long-term (≥ 5 years of follow-up) prognostic usefulness of coronary CTA.

## Introduction

Coronary CT angiography (CTA) has emerged as a highly accurate non-invasive test for the diagnosis of coronary artery disease (CAD). Iterative improvements in CT scanner technology and imaging protocols have resulted in significantly lower patient radiation and iodinated contrast exposure with improved image quality. Supporting its clinical utilization, numerous contemporary clinical guidelines and appropriate use criteria recommend cardiac CT for a variety of clinical indications [1–3]. Coronary CTA is unique among non-invasive tests based on its ability to accurately visualize and quantify the presence and extent of both angiographically significant and non-obstructive coronary atherosclerosis. Furthermore, as a 3-dimensional imaging modality, coronary CTA provides detailed coronary plaque characteristics that have been shown to provide important prognostic information beyond that obtained from stenosis severity and location alone. Highlighting the potential of coronary CTA for improving patient outcomes, randomized prospective trials have shown that patients undergoing coronary CTA have significantly reduced risk of incident myocardial infarction, potentially related to the increased utilization of preventive therapies (e.g., statins) among patients with non-obstructive CAD, as compared to patients who underwent functional testing for CAD [4•]. In the current era of value-based imaging, it is imperative that tests for CAD not only be accurate, but that they also inform patient and provider therapeutic and preventive interventions through the refinement of individual cardiovascular prognosis, beyond that provided by risk factors alone. Herein, we review recent large-scale studies that have evaluated the long-term (defined as > 5 years of follow-up) prognostic value of coronary CTA and we discuss the implications of these data on patient management.

## Coronary CTA and prognosis: lessons learned from early studies

Early studies evaluating the short-term prognostic implications of coronary CTA findings typically utilized worst lumen stenosis as the primary measure of CAD severity [5]. In an initial large-scale meta-analysis, Hulten and colleagues demonstrated that among 9592 subjects who underwent coronary CTA who were followed for a median of 20 months (mean age 59 years; 58% men), the presence of angiographically significant (> 50% stenosis) CAD was associated with 3.2% incidence of death or myocardial infarction (MI) as compared to 0.15% in those without CAD (*p* < 0.05) [6]. Similarly, patients with non-obstructive CAD had a 1.4% annualized risk of death, MI, or coronary revascularization, which was significantly higher than the 0.15% annualized risk for this combined endpoint seen in patients with a normal CTA. This early meta-analysis highlighted the prognostic importance of both angiographically significant (potentially flow-limiting) and non-obstructive coronary stenosis (< 50%), as well as the excellent prognosis for patients without CAD evident on coronary CTA. Moving beyond stenosis, shorter-term studies also confirmed that the extent of CAD, as measured simply by the number of coronary arterial segments with plaque (segment involvement score, SIS) and location of stenosis (left main, proximal left anterior descending [LAD]) strongly predicted incident mortality and myocardial infarction and may determine optimal management [7, 8]. More recently, a meta-analysis of 9777 subjects confirmed the prognostic importance of the SIS [9•]. Specifically, the SIS (per segment increase) was associated with a pooled HR of 1.25 (95% confidence interval [CI] 1.16–1.35; *p* < 0.001) for incident major adverse cardiovascular events (MACE, death, MI, or late coronary revascularization). Segment stenosis (per segments with > 50% stenosis) was associated with a pooled HR of 1.37 (1.32–1.42) and any stenosis > 50% (binary) was associated with a pooled HR of 3.39 (1.65–6.99) for MACE.

Demonstrating the potential implications of prognostic findings on coronary CTA on management, in an important analysis from the large-scale, multinational CONFIRM (Coronary CT Angiography Evaluation For Clinical Outcomes) registry, investigators demonstrated that among 15,223 patients without known CAD who underwent coronary CTA, only those with high-risk CAD, defined as 2-vessel CAD (> 50% stenosis) involving the proximal LAD, 3-vessel or left main CAD, gained a mortality benefit from subsequent coronary revascularization [10]. Highlighting the prognostic importance of high-risk plaque features, Motayama and colleagues followed 1059 patients with non-obstructive CAD on coronary CTA over a mean of 27 months [11]. Within this cohort, the presence of CAD with both positive (expansile) remodeling (PR) and low-attenuation plaque (LAP, defined as any plaque component < 30 Hounsfield units [HU]) was associated with more than a 20-fold increased likelihood of subsequent acute coronary syndrome (ACS) (HR 22.8; 6.9–75.2). In addition, coronary plaques that were shown to be culprit lesions in future ACS events were noted to have an increased remodeling index (RI 1.27 vs 1.13; *p* = 0.003), total plaque volume (134.9 vs 57.8 mm^3^; *p* < 0.001) and LAP volume (20.4 vs 1.1 mm^3^; p < 0.001). Subsequent investigators have further identified other high-risk or vulnerable plaque characteristics that imply an increase patient risk for MACE. In addition to those mentioned above, these features include spotty calcification (<3 mm diameter), increased lesion length, total coronary plaque burden, and the presence of the napkin ring sign (NRS), generally defined as peripheral plaque enhancement surrounding lower attenuation plaque.

These studies reviewed above, involving short- and intermediate-term follow-up, provide an important foundation to understanding longer-term studies reporting the prognostic value of coronary CTA. In addition, they provide the rationale for many of the CT-based risk scores utilized in assessing long-term prognosis. In the following section, we review and summarize studies published within the past 2 years reporting long-term (defined herein as ≥ 5 years of follow-up) outcomes following coronary CTA.

## Recent studies of long-term prognosis

One of the initial studies to report the long-term prognostic importance of coronary CTA was published in 2013 by Hadamitzky and colleagues [12]. In this study, the severity of CAD stenosis (> 50%) and the total coronary plaque score (number of coronary segments with ≥ 25% stenosis) were the best predictors of death and non-fatal MI, with both measures significantly improving predictive accuracy as compared to standard clinical risk scores (multivariate c-index 0.60 and 0.66, respectively, *p* = 0.002 and < 0.0001, respectively). Consistent with shorter-term studies, CAD stenosis and segmental involvement revealed important differences in prognosis. For example, the annualized event rate ranged from 0.24% for patients with no CAD to 1.1% for patients with obstructive (> 50% stenosis) CAD and 1.5% for patients with CAD and extensive plaque involvement (> 5 segments). In Table 1 we summarize subsequent studies published within the past 2 years that reported the long-term (≥ 5 years of follow-up) prognostic implications of coronary CTA findings. As compared to earlier studies involving shorter follow-up durations, recently published long-term studies confirm the prognostic value of stenosis, but further highlight the prognostic importance of both plaque characteristics and risk scores that are derived from coronary CTA studies.

**Table 1:** Selected recent (2016–present) studies reporting long-term (at least 5 years) prognosis following coronary CT angiography

Main author	Year	Design	Patient population (known or suspected CAD)	Sample Size (*n*)	Mean follow-up (years)	Mean age	Outcome measures	CTA measures	Results
Feuchtner [13]	2017	PCO	Suspected	1469	7.8	65.9	1^o^–MACE (MI or UA) 2^o^–Coronary revascularization	1. Stenosis severity 2. Plague types 3. High-risk plague criteria(LAP < 60 HU, NRS, SC, and RI)	1. Excellent long-term prognosis if CTA is negative 2. LAP < 60HU and NRS powerful MACE predictors with HR 4.96 and 3.85, respectively
Nadjiri [14]	2016	PCO	Suspected	1168	5.7	58.6	MACE (cardiac death, MI and late revascularization)	Plaque characteristics: LAP < 30HU, NCPV, RI, and NRS	LAP < 30HU strongest predictor for MACE, HR 1.12. LAP additive to Morise score, CACS, and SSS (*p* = 0.036)
Cheruvu [15]	2016	PCO	Suspected	1884	5.6	55.6	1^o^–All-cuase mortality 2^o^–MACE (combination of death, non-fatal MI, UA and late revascularization)	Stenosis severity: none (0% stenosis); non-obstructive (1 to 49% stenosis); obstructive (≥ 50% stenosis)	Mortality: non-obstructive: HR 1.73 (1.07–2.79) Obstructive 1 and 2 vessel: HR 1.70 (1.08–2.71 3. Vessel/LM: HR 2.87 (1.57–5.23). MACE: non-obstructive HR 2.20 (1.31–3.67); obstructive HR 6.63 (3.91–11.26)
Blanke [16]	2016	PCO. Propensity-score matched (1:1) diabetics (1823) vs non-diabetic patients (1823)	Known	3646	5.0	61.7	1^o^–All-cause mortality 2^o^–MACE (death, MI, UA, or late revascularization)	Stenosis severity: none (0% stenosis); non-obstructive (1 to 49% stenosis); obstructive (≥ 50% stenosis)	Diabetes and no CAD: no difference in mortality (HR 1.32; 0.78–2.24; *p* = 0.296). Diabetes and non-obstructive CAD (HR 2.09; 1.43–3.09) and obstructive CAD (HR 1.95; 1.46–2.61) as compared to non-diabetic patients MACE significantly increased (assessed only among 973 diabetics)
Nadjiri [17]	2016	PCO	Suspected	1487 (108 with diabetes)	5.3	65	Mortality, non-fatal MI, or UA requiring hospitalization	CACS, SIS, SSS	In diabetic patients, SIS and SSS showed significant prognostic value over Framingham score with HR of 2.98 and 4.47, respectively
Deseive [18]	2017	PCO	Suspected	15,219	5.0	58.7	All-cause mortality	CONFIRM risk score	CONFIRM score outperformed Morise score and risk-factor based scores: c-index .696
Andreini [19]	2017	PCO	Suspected CAD with non-obstructive (< 50%) stenosis	2402	5.0	56	Non-fatal MI and MI + all-cause mortality	SIS, plaque composition, and CT-adapted LeSc	LeSc strongest predictor of MI (HR 2.84) and MI + death (HR 2.48). LeSc superior to SIS, plaque measures, and risk factors.

Abbreviations: *CACS* coronary artery calcium score, *CAD* coronary artery disease, *CTA* CT angiography, *HR* hazards ratio, *HU* Hounsfield unit, *LAP* low-attenuation plaque, *LeSc* Leaman score, *LM* left main, *MACE* major adverse cardiovascular events, *NCPV* non-calcified plaque volume, *NRS* napkin ring sign, *PCO* prospective cohort, *RetCO* retrospective cohort, *RI* remodeling index, *SC* spotty calcification, *SIS* segment involvement score, *SSS* segment stenosis score, *UA* unstable angina

**Fig. 1:**
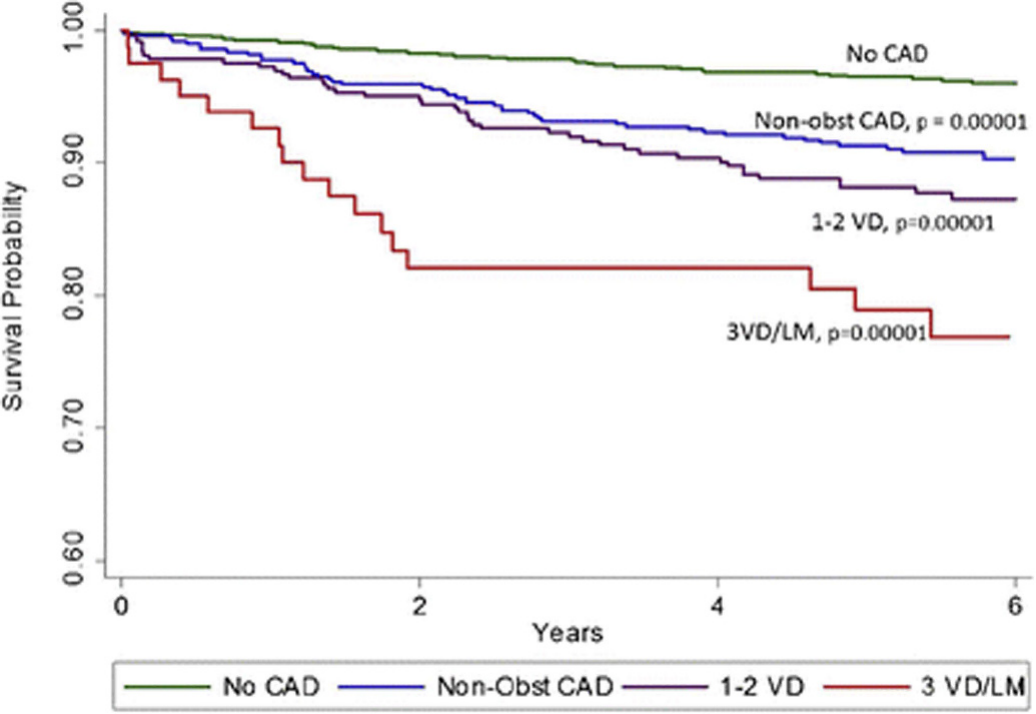
Unadjusted Kaplan-Meier curve for mortality-free survival on the basis of the presence of no CAD, non-obstructive CAD, 1- and 2-vessel obstructive CAD and 3-vessel obstructive and left main CAD for individuals without modifiable CAD risk factors (*p* values based on log-rank tests). (*Reused with permission from Elsevier*) [15].

Introduced above, the CONFIRM registry has served as a foundational large-scale cohort from which the prognostic value of coronary CTA has been established. With the extension of the registry to allow for long-term (> 5 year) follow-up, several recent reports from CONFIRM have provided valuable insights on this topic. For example, the findings, mentioned above, that only those with high-risk CAD on coronary CTA derive a benefit from revascularization has been extended and confirmed among CONFIRM registry patients with more than 5.5 years of follow-up [20]. Additionally, Cheruvu and colleagues studied 1884 patients without prior CAD or any modifiable CAD risk factors [15]. Among this low-risk group (mean age 55.6 years) followed for a mean of 5.6 ± 1.3 years, the presence of > 1 segment of non-obstructive CAD (HR 1.73; 1.08–2.71), obstructive (> 50%) 1- or 2-vessel CAD (HR 1.7; 1.08–2.71) and 3-vessel or left main obstructive CAD (HR 2.87; 1.57–5.23) were significantly associated with increased all-cause mortality (Fig. 1). Similarly, the incidence of MACE (all-cause death, non-fatal MI, unstable angina, or late coronary revascularization) increased from 5.6% in those without CAD to 13.24% in those with non-obstructive disease and to 36.28% in those with obstructive CAD (*p* < 0.001). These results highlight the prognostic implication of manifest CAD on coronary CTA among patients without cardiovascular risk factors, and the potential of these findings to refine management. For example, young, low-risk patients are frequently not offered preventive therapies for primary cardiovascular prevention, such as aspirin or statins, based on global risk scores. Also from CONFIRM, Blanke and colleagues compared outcomes between 1823 patients with diabetes to 1823 propensity-matched patients without diabetes who were followed for 5 years following coronary CTA for incident mortality [16]. Highlighting the impact of CAD burden on the prognosis of diabetic patients, patients with diabetes did not exhibit an increased risk of mortality compared with the propensity-matched non-diabetic subjects in the absence of CAD (HR 1.32; 0.78–2.24; *p* = 0.296). However, among subjects with non-obstructive CAD on CTA, diabetic patients were at significantly increased risk of death (HR 2.10; 1.43–3.09) with a mortality risk that was higher than non-diabetic patients with obstructive (≥ 50% stenosis) CAD. These data highlight the clinical implications of CAD, both obstructive and non-obstructive, when present among diabetic patients, and are similar to other smaller long-term studies [17, 21]. While these 2 reports from CONFIRM are observational in nature, we feel that they support intensification of preventive therapy following CTA in the presence of CAD and should also be incorporated into patient counseling regarding cardiovascular risk and lifestyle modifications.

The CONFIRM investigators also recently assessed the long-term prognostic impact of several risk scores that attempt to quantify CAD severity as assessed on coronary CTA. Deseive and colleagues reported long-term mortality from among 15,219 patients within the CONFIRM registry according to the CT-derived CONFIRM risk score [18]. The CONFIRM score was originally described in 2013 [22]. In this original report, investigators demonstrated that compared to plaque composition (non-calcified, partially-calcified, or calcified) and measures of stenosis severity and location, the best CCTA parameter for prediction of mortality over 2.3 years was the number of proximal segments with mixed or calcified plaques (c-index 0.64, *p* < 0.0001) and the number of proximal segments with a stenosis >50% (c-index 0.56, *p* = 0.002). Using the CONFIRM score, that included both parameters, coronary CTA significantly improved overall risk prediction beyond traditional risk factors and risk-factor scores. Similar to the original shorter-term analysis, over a median of 5.3 years of follow-up, the CONFIRM score outperformed three clinical risk scores (Framingham, Morise score, and NCEP ATP III) for the prediction of long-term all-cause mortality.

CONFIRM investigators recently examined the prognostic value of the CT-adapted Leaman score (CT-LeSc), a score initially derived from invasive coronary angiography. The CT-LeSc incorporates coronary CTA information regarding lesion location, stenosis severity, and plaque composition. In an earlier study, Mushtaq and colleagues evaluated 1304 consecutive patients undergoing coronary CTA for suspected coronary artery disease and reported atherosclerotic scores (segment involvement score [SIS], segment stenosis score [SSS]), and CT-LeSc [23]. Patients were followed over a mean follow-up of 52 ± 22 months for the primary outcome of cardiac death and ACS. While all scores were independent predictors of cardiac events (HR 3.09 for SIS, 4.42 for SSS, and 5.39 for CT-LeSc), the investigators concluded that the CT-LeSc was the strongest independent long-term predictor of hard cardiac events. Among subjects with non-obstructive CAD, patients with a high CT-LeSc (score > 5) had an event-free survival comparable to patients with obstructive disease. Cumulative event-free survival was 76.8% in patients with CT-LeSc > 5 and 96.0% with CT-LeSc ≤ 5. Similarly, within CONFIRM, Andreini and colleagues recently demonstrated that among 2402 patients without prior CAD and non-obstructive CAD on coronary CTA followed over a mean of 60 months, the CT-LeSc was the strongest predictor of incident MI and all-cause mortality as compared to traditional risk factors and other CT measures of CAD severity, to include the SIS [19].

Highlighting the potential impact of plaque morphology on prognosis, Feuchtner and colleagues evaluated 1469 low-to-intermediate risk patients with coronary CTA for stenosis severity (minimal < 10%; mild < 50%; moderate 50–70%; severe >70%), plaque type (calcified, mixed dominantly calcified, mixed dominantly non-calcified, non-calcified), and high-risk plaque criteria (LAP, NRS, spotty calcification < 3 mm, and RI) [13]. Over 7.8 years of follow-up, the presence of LAP (< 60 HU) and the NRS were the strongest predictors of incident MACE (adjusted HR 4.96 and 3.85, respectively; *p* < 0.0001), with stenosis > 50% (HR 1.5; 1.1–2.3) and increased non-calcified plaque component (HR 1.7; 1.1–2.6) also predictive in adjusted models. In a similar analysis of plaque characteristics, Nadjiri and colleagues assessed 1168 consecutive patients with suspected CAD [14]. During 5.7 years of follow-up, cardiac death, MI, or coronary revascularization occurring > 90 days after CTA occurred in 3.9% of subjects. Compared to the NRS and SSS, the strongest association with incident MACE was for low-attenuation plaque volume (HR 1.12, *p* < 0.0001) as measured using semi-automated techniques. Addition of low-attenuation plaque volume to the Morise score, coronary artery calcium score and clinical variables slightly improved risk prediction.

## Clinical implications

Short- and long-term studies evaluating the clinical importance of coronary CTA measures of coronary atherosclerosis have clearly demonstrated that the presence, location, and severity of CAD impart powerful prognostic information. Measures of both stenosis severity and plaque features on CTA appear to be superior to both coronary artery calcium scoring and traditional cardiovascular risk factors, while the absence of coronary atherosclerosis on high-resolution CTA images identifies a patient with an exceptionally low risk of long-term cardiovascular events [24, 25]. As a result, patients and providers should modify the intensity of preventive therapies accordingly [26•]. Accordingly, in 2015, the Society of Cardiovascular Computed Tomography published the Coronary Artery Disease Reporting and Data System (CAD-RADS™) whereby coronary CTA results are systematically linked to management recommendations [27••]. For example, among patients with non-obstructive CAD (especially involving multiple segments), providers should strongly consider preventive therapies, such as aspirin and statins, in addition to therapeutic lifestyle changes. Among patients with high-risk (left main, 3-vessel or proximal LAD) obstructive CAD, consideration for ICA should be considered; among those with lower-risk but potentially flow-limiting CAD, functional testing and guideline-directed medical therapy should be considered. Importantly, CAD-RADS™ incorporates spotty calcification, PR, NRS, and LAP (< 30 HU) as prognostically important measures of plaque vulnerability that should be included in the CTA report, when present. This approach may serve to not only standardize CTA reporting, but also link CTA findings to appropriate changes in management that may improve patient outcomes and overall imaging value.

While no randomized controlled trials have been performed specifically to assess the impact of coronary CTA-guided preventive therapy, observational studies and clinical trials suggest that the intensification of statin and other preventive therapy may improve outcomes. The visualization of CAD on CTA has been repeatedly shown to result in the initiation of statin and aspirin therapy in large-scale observational studies [28, 29]. Hwang and colleagues prospectively followed 8372 patients (mean age 61 years old) with non-obstructive CAD on coronary CTA over a mean of 828 days for all-cause mortality and the combination of mortality and late (> 90 days) coronary revascularization [30•]. Subjects who were initiated on a statin following CTA (23.7%) had significantly lower risk for death and the combined outcome, adjusted HR 0.40 (0.26–0.60) and HR 0.43 (0.31–0.60), respectively. Similarly, in CONFIRM subjects with either normal (*n* = 5712) or non-obstructive CAD (*n* = 4706) followed over 27 months, statin use at the time of coronary CTA was independently associated with a reduced risk of mortality (adjusted HR 0.44, 0.28–0.68; *p* = 0.0003), but only among subjects with CAD [31]. Finally, recent comparative effectiveness studies have shown that, compared to functional tests, patients undergoing coronary CTA are more likely to be prescribed preventive therapies, translating into potentially improved outcomes. In a post-hoc analysis of the SCOT-HEART (Scottish Computed Tomography of the HEART) randomized comparative effectiveness trial (*n* = 4173), patients who underwent CCTA were noted to have a subsequent 4-fold increase in the use of statins and aspirin therapy as compared to the functional testing arm [32••]. This significant change in post-test management was associated with a 50% reduction (HR 0.50; 0.28–0.88; *p* = 0.020) in incident fatal and non-fatal MIs over just 1.7 years of follow-up.

Despite the robustness of the data presented above, significant additional work is needed to further refine and better standardized the prognostic information garnered from coronary CTA. First, as highlighted in the studies above, significant variability exists as how to best quantify risk from a coronary CTA dataset. Stenosis severity and plaque characteristics have been most commonly assessed, in part, due to their ease of use. However, numerous, somewhat similar CAD “scores” exist, many of which are not commonly utilized. Recent studies suggest that more detailed measurements of overall and non-calcified plaque volume may incrementally improve the prognostic value of coronary CTA. While these three-dimensional measures of coronary atherosclerosis are difficult to quantify manually, automated and semi-automated calculation of total and non-calcified plaque volume may allow for a broader clinical utilization in patient-level decision making. Not surprisingly, investigators have demonstrated that lesions with increased plaque volume are more likely to be related to an abnormal invasive fractional flow reserve and abnormalities on cardiac PET imaging [33–35]. Longer-term prognostic studies assessing measures of plaque burden are emerging and will only enhance our knowledge in this area [36]. Additionally, machine learning may allow for a more precise assessment of cardiovascular risk through more accurate incorporation of a broader array of clinical and CT-based variables [37]. Finally, the measurement of CT-derived FFR may improve decision making following coronary CTA, but may also provide additional prognostic information beyond stenosis and measures of plaque vulnerability [34]. Further studies on the long-term prognostic value of CT-derived FFR are of clinical interest. Ultimately, it is imperative that providers understand the long-term prognostic implications of commonly reported coronary CTA findings and ensure that appropriate preventive and therapeutic measures, as well as patient lifestyle coaching, are instituted accordingly. Only in this manner will we maximize the value of coronary CTA to patients, providers, and the healthcare system.
